# Supplementary material for: Application of Cold Storage and Short In Vitro Germination for Somatic Embryos of Pinus radiata and P. sylvestris
Source: Plants (Basel). 2023 May 24;12(11):2095. doi: 10.3390/plants12112095 (PMC10255715; doi:10.3390/plants12112095)

Table S1. Percentage survival of emblings originated from all tested *Pinus radiata* cell lines and treatments at the repotting stage and at the end of the experiment (Final), n = number of plantlets originally planted in each treatment/cell line combination. Note that survival final is based on the number of emblings which were transferred to their final pot (at repotting), for control this was when they went from the propagation tray to the final container, for Trt 1 (short germination/micro-plug protocol) it was when they went from the micro-plug to the final container and for Trt 2 (cold storage/short germination protocol) it was when they went from the germination medium to final container.

**Origin of emblings/ cell Line**	**Percentage of survival (Repotting)**				**Percentage of survival (Final)**					
	**n**	**Control**	**n**	**Treatment 1**	**n**	**Control**	**n**	**Treatment 1**	**n**	**Treatment 2**
A	30	66.67	27	62.96	30	40.00	27	75.00	30	53.57
B	28	75.00	30	56.67	28	47.62	30	41.18	28	40.00
C	46	30.43	60	85.00	46	100.00	60	82.35	45	22.22
D	71	61.97	30	70.00	71	61.36	30	100.00	45	5.00
E	10	90.00	10	80.00	10	66.67	10	87.50	45	35.56
F	10	80.00	10	80.00	10	62.50	10	100.00	45	72.50
G	11	100.00	10	50.00	11	54.55	10	100.00	45	43.33
H	16	87.50	20	90.00	16	100.00	20	94.44	45	62.50
I	33	93.94	25	84.00	33	54.84	25	100.00	45	25.00
J	35	91.43	20	90.00	35	34.38	20	100.00	45	30.00
K	61	75.00	25	92.00	61	57.78	25	82.61		
L	25	47.22	25	96.00	25	78.79	25	75.00		
M	20	100.00	20	90.00	20	68.42	20	100.00		
N	34	23.08	34	100.00	34	80.00	34	82.35		
O	36	77.27	36	88.89	36	8.82	36	84.38		
P	48	92.59	48	95.83	48	78.00	48	80.43		
Q	**48**	49.15	48	97.92	**48**	58.62	48	85.11		
R	34	51.52	34	88.24	34	58.82	34	90.00		

Table S2. Average shoot height, root length and root collar diameter and standard deviations of surviving *Pinus radiata* emblings that originated from all tested cell line in each of the 3 treatments. Different letters indicate significant differences among treatments for each cell line.

Origin of emblings/ cell line	Shoot height ± SD (mm)			Root length ± SD (mm)			Root collar diameter ± SD (mm)		
	Control	Trt 1	Trt 2	Control	Trt 1	Trt 2	Control	Trt 1	Trt 2
A	17.88 ± 8.37 (b)	61.23 ± 25.81 (a)	31.27 ± 11.66 (a)	110.75 ± 26.42 (a)	124.15 ± 42.60 (a)	107.20 ± 14.98 (a)	0.93 ± 0.15(b)	1.12 ± 0.21(a)	1.13 ± 0.26 (a)
B	28.40 ± 14.21(b)	72.29 ± 22.88 (a)	26.90 ± 15.82 (b)	127.00 ± 34.75 (a)	162.57 ± 41.22 (a)	112.80 ± 24.73 (b)	1.19 ± 0.42 (a)	1.23 ± 0.12(a)	1.31 ± 0.32 (a)
C	27.21 ± 17.01(b)	44.02 ± 26.25 (a)	37.10 ± 15.34 (a)	70.79 ± 31.40 (a)	93.93 ± 44.67 (a)	87.20 ± 23.73 (a)	1.05 ± 0.28 (a)	1.03 ± 0.31 (a)	0.90 ± 0.13 (a)
D	32.38 ± 13.80(b)	68.24 ± 21.3 (a)	24.00 ± 5.66 (b)	118.15 ± 35.52 (a)	128.14 ± 40.90 (a)	106.00 ± 1.41 (a)	1.00 ± 0.19(b)	1.45 ± 0.31(a)	1.04 ± 0.23 (b)
E	35.66 ± 18.45 (b)	67.00 ± 18.39(a)	62.88 ± 22.72 (a)	103.00 ± 23.14 (a)	131.6 ± 34.18 (a)	140.94 ± 40.06 (a)	1.17 ± 0.39 (a)	1.24 ± 0.33 (a)	1.44 ± 0.37 (a)
F	63.00 ± 15.30 (b)	128.63 ± 9.33(a)	77.86 ± 26.33 (b)	151.20 ±47.67 (a)	169.38 ± 25.72 (a)	120.45 ± 27.40 (b)	1.46 ± 0.14 (b)	1.68 ± 0.06 (a)	1.35 ± 0.28 (b)
G	33.67 ± 28.62 (b)	83.00 ± 24.7 (a)	46.69 ± 18.84 (b)	94.00 ± 45.37 (b)	155.4 ± 35.78 (a)	95.77 ± 21.56 (b)	1.03 ± 0.41 (a)	1.33 ± 0.20 (a)	1.06 ± 0.31 (a)
H	55.14 ± 19.57 (b)	89.47± 32.77 (a)	61.80 ± 27.45 (b)	126.94 ± 34.05 (a)	122.35 ± 28.73 (a)	107.22 ± 26.83 (b)	1.34 ± 0.22 (a)	1.39 ± 0.36 (a)	1.00 ± 0.28 (b)
I	31.94 ± 11.4 (b)	81.24 ± 25.09 (a)	43.00 ± 17.17 (b)	126.94 ± 34.05 (b)	159.38 ± 44.83 (a)	127.20 ± 31.37 (b)	1.22 ± 0.20 (a)	1.32 ± 0.19 (a)	1.20 ± 0.18 (a)
J	48.73 ± 16.79 (b)	91.11 ± 31.92 (a)	30.67 ± 16.21 (b)	143.45 ± 31.93 (a)	136.61 ± 46.48 (a)	97.00 ± 28.00 (b)	1.21 ± 0.21(a)	1.29 ± 0.33 (a)	0.99 ± 0.39 (b)
K	39.08 ± 20.71 (b)	92.84 ± 15.60 (a)		112.35 ± 33.38 (b)	149.74 ± 21.03 (a)		1.36 ± 0.38 (b)	1.59 ± 0.19(a)	
L	54.58 ± 21.86 (b)	96.50 ± 18.24 (a)		112.89 ± 25.95 (b)	135.06 ± 20.35 (a)		1.34 ± 0.34 (b)	1.82 ± 0.20 (a)	
M	60.54 ± 20.81 (b)	101.50 ± 23.43 (a)		116.31 ± 22.83 (b)	142.39 ± 20.32 (a)		1.36 ± 0.28 (a)	1.42 ± 0.17 (a)	
N	71.17 ± 30.54 (a)	72.36 ± 24.58 (a)		111.67 ± 27.10 (a)	113.39 ± 19.33 (a)		1.11 ± 0.32 (a)	1.14 ± 0.18 (a)	
O	39.67 ± 7.57 (a)	78.33 ± 34.14 (a)		60.00 ± 31.22 (b)	108.22 ± 21.39 (a)		1.03 ± 0.34 (a)	1.19 ± 0.23 (a)	
P	65.05 ± 25.46 (b)	90.05 ± 24.36 (a)		99.80 ± 29.20 (b)	130.97 ± 20.83 (a)		1.14 ± 0.23 (b)	1.31 ± 0.17 (a)	
Q	50.00 ± 31.75 (b)	76.90 ± 25.58 (a)		79.35 ± 37.91 (b)	117.10 ± 18.07 (a)		0.96 ± 0.23 (b)	1.13 ± 0.20 (a)	
R	75.1 ± 26.31 (a)	72.92 ± 23.38 (a)		107.30 ± 21.88 (a)	127.11 ± 28.36 (a)		1.17 ± 0.21 (a)	1.14± 0.19 (a)	

Figure S1. Length of *Pinus sylvestris* germinants from four cell lines in two treatments (Control and MB 2w) after two weeks of *in vitro* germination (shoot in positive axis, root in negative axis) in the Dec20 trial. Mean values ± SEM.

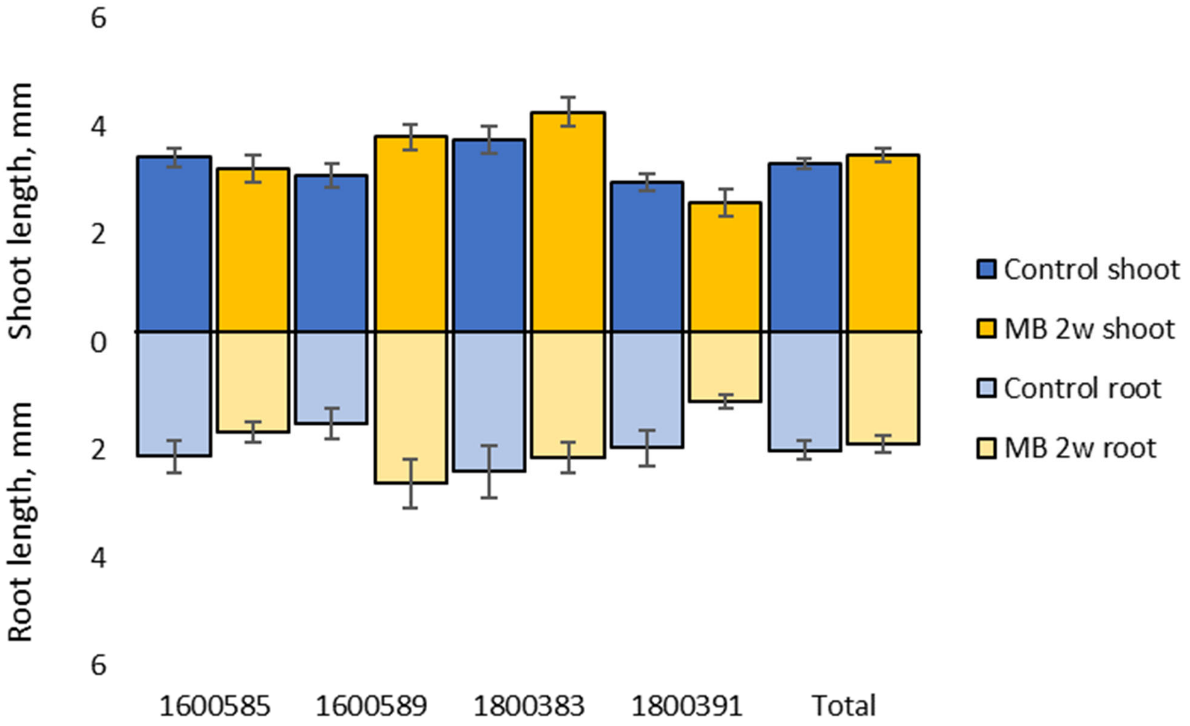

Figure S2. Length of *Pinus sylvestris* germinants from seven cell lines in three treatments (Control, MB 2w and LM 2w) after two weeks of *in vitro* germination (shoot in positive axis, root in negative axis) in the Jan21 trial. Mean values ± SEM.

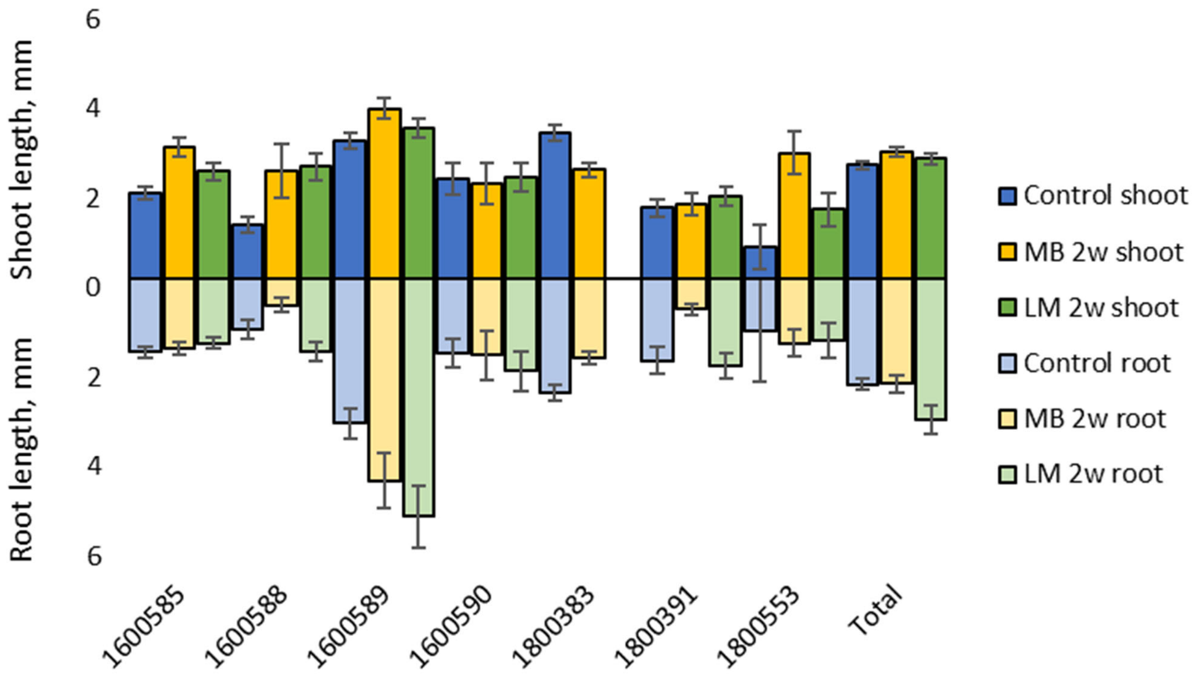

Supplement: Supplementary file 1 [file plants-12-02095-s001.zip › plants-2373296-supplementary.pdf]
